# Efficacy and Safety of Intrathecal Buprenorphine for Postoperative Analgesia in Cesarean Delivery: A Systematic Review of Randomized Controlled Trials

**DOI:** 10.7759/cureus.107092

**Published:** 2026-04-15

**Authors:** Luc G Corriveau

**Affiliations:** 1 College of Nursing: Nurse Anesthesiology, University of Arizona, Tucson, USA

**Keywords:** analgesia, buprenorphine, buprenorphine safety profile, cesarean section, enhanced recovery after surgery (eras), intrathecal buprenorphine, intrathecal opioids, multimodal analgesia, opioid-sparing analgesia, postoperative pain management

## Abstract

Cesarean delivery is one of the most commonly performed surgical procedures worldwide and is most commonly conducted under neuraxial anesthesia when not contraindicated. Intrathecal morphine remains the most commonly used neuraxial opioid adjunct for cesarean delivery and is widely considered the standard of care due to its prolonged duration of postoperative analgesia. However, neuraxial morphine is associated with dose-dependent adverse effects, including pruritus, nausea, urinary retention, and delayed respiratory depression related to cephalad cerebrospinal fluid migration. Buprenorphine, a semi-synthetic opioid derived from thebaine, functions as a partial μ-opioid receptor agonist with high receptor affinity and slow dissociation kinetics, potentially offering prolonged analgesia with a favorable safety profile. This systematic review evaluates the efficacy and safety of intrathecal buprenorphine for postoperative analgesia in cesarean delivery. The review was conducted in accordance with the Preferred Reporting Items for Systematic Reviews and Meta-Analyses (PRISMA 2020) guidelines. PubMed and the Cochrane Central Register of Controlled Trials (CENTRAL) were searched from inception through February 22, 2026. Randomized controlled trials (RCTs) involving women undergoing cesarean delivery under spinal anesthesia comparing intrathecal buprenorphine with local anesthetic alone or alternative intrathecal regimens were included. Primary outcomes were duration of analgesia and time to first rescue analgesic. Secondary outcomes included pain scores and adverse events. Risk of bias was assessed using the Cochrane Risk of Bias 2 tool. Five randomized controlled trials involving 727 participants met the inclusion criteria. Intrathecal buprenorphine consistently prolonged postoperative analgesia and delayed time to rescue analgesic compared with the control groups. A dose-dependent pattern was observed, supported by both within-trial dose comparisons and consistent trends across studies. Analgesic duration ranged from approximately 173 minutes to 18 hours, with lower doses (≤60 µg) associated with shorter durations and higher doses (≥100 µg) producing more prolonged analgesia. Adverse events were generally mild and included nausea, vomiting, pruritus, and sedation, with higher doses associated with increased frequency of these effects. No clinically significant respiratory depression was reported in any included trial; however, reporting of respiratory monitoring protocols was inconsistent across studies. Intrathecal buprenorphine appears to be an effective and generally safe adjuvant for post-cesarean analgesia. Lower doses may provide a clinically favorable balance between analgesic efficacy and tolerability. However, heterogeneity in dosing strategies, comparator regimens, and outcome definitions, as well as small sample sizes, limits certainty. Larger high-quality randomized trials directly comparing buprenorphine with intrathecal morphine are warranted.

## Introduction and background

Cesarean delivery represents one of the most frequently performed surgical procedures globally and is commonly conducted under neuraxial anesthesia when not contraindicated [1]. Effective postoperative analgesia following cesarean delivery is essential to facilitate early mobilization, maternal-neonatal bonding, and breastfeeding while minimizing systemic opioid exposure, and is a key component of enhanced recovery after surgery (ERAS) protocols in obstetric practice.

Intrathecal morphine remains the most commonly used neuraxial opioid adjunct for cesarean delivery and is widely regarded as the standard of care due to its prolonged duration of postoperative analgesia [2]. However, neuraxial morphine is associated with dose-dependent adverse effects, including pruritus, nausea, urinary retention, and delayed respiratory depression resulting from cephalad cerebrospinal fluid migration [2].

Buprenorphine is a semi-synthetic opioid derived from thebaine that functions as a partial μ-opioid receptor agonist with high receptor affinity and slow dissociation kinetics [3]. Its affinity for the μ receptor exceeds that of morphine, contributing to prolonged duration of action and relative resistance to antagonism with naloxone [3]. Its high lipid solubility and receptor-binding characteristics suggest potential for sustained spinal analgesia with reduced rostral spread, although cephalad migration may still occur, particularly at higher doses.

Several randomized trials have evaluated intrathecal buprenorphine in cesarean delivery, but variability in dosing strategies, comparator regimens, and outcome definitions has limited clarity regarding its clinical role [4-8]. A structured synthesis of randomized evidence is necessary to determine its efficacy and safety profile.

The objective of this systematic review was to evaluate the efficacy and safety of intrathecal buprenorphine for postoperative analgesia in women undergoing cesarean delivery under spinal anesthesia.

## Review

Methods

Protocol and Reporting Standards

This systematic review was conducted in accordance with the Preferred Reporting Items for Systematic Reviews and Meta-Analyses (PRISMA 2020) guidelines [9]. A PRISMA 2020 checklist was completed to ensure reporting compliance. A protocol was developed internally prior to study selection to define eligibility criteria, outcomes, and analytic strategy; however, the review was not prospectively registered in a public database, as it was conducted as an exploratory synthesis of available randomized evidence.

Search Strategy

Electronic searches were conducted in PubMed (via NCBI) and the Cochrane Central Register of Controlled Trials (CENTRAL), with the final search performed on February 22, 2026. The PubMed search strategy combined Medical Subject Headings (MeSH) and free-text terms related to buprenorphine, spinal anesthesia (including intrathecal administration), and cesarean delivery, along with filters for randomized controlled trials. Similarly, the CENTRAL search strategy used combinations of keywords including buprenorphine, intrathecal or spinal anesthesia, and cesarean delivery to identify relevant studies.

Eligibility Criteria

Studies were included if they were randomized controlled trials involving adult women undergoing cesarean delivery under spinal anesthesia in which intrathecal buprenorphine was administered as an adjuvant to local anesthetic and compared with either local anesthetic alone or alternative intrathecal or epidural opioid regimens, with reporting of postoperative analgesic outcomes. Studies were excluded if they were non-randomized or observational in design, did not involve cesarean delivery, lacked a comparator group, or were case series, abstracts without full data, reviews, or editorials. No date or language restrictions were applied. Non-English articles were screened, and translation using artificial intelligence-assisted tools (ChatGPT, OpenAI, San Francisco, CA, US) was utilized where necessary during the screening process; however, none met the predefined inclusion criteria and were therefore not included in the final analysis, with all eligibility decisions subsequently confirmed by the author and an independent reviewer. Studies identified in the search that did not meet predefined inclusion criteria (e.g., differences in study design, population, or outcome reporting) were excluded. Clinical trial registries and unpublished data sources were not systematically searched, and no unpublished studies were included in this review.

Study Selection

Two reviewers independently screened titles and abstracts for eligibility, and full texts of potentially relevant articles were subsequently assessed independently by both reviewers, with disagreements resolved through consensus discussion. No automation tools were used for screening.

Data Extraction

Data extraction was performed independently by two reviewers using a standardized extraction form that captured study design, sample size, buprenorphine dose, comparator group, duration of analgesia, time to first rescue analgesic, postoperative pain scores, and adverse events. Discrepancies were resolved through consensus discussion, and study authors were not contacted for additional data.

Risk of Bias Assessment

Risk of bias was assessed at the outcome level using the Cochrane Risk of Bias 2 (RoB 2) tool [10], evaluating domains including bias arising from the randomization process, deviations from intended interventions, missing outcome data, measurement of outcomes, and selection of reported results. Assessments were performed independently by two reviewers, with disagreements resolved by consensus.

Data Synthesis

Due to heterogeneity in outcome definitions and reporting, as well as variability in buprenorphine dosing strategies and comparator regimens, a quantitative meta-analysis was not performed. Studies were grouped according to dosing strategy and comparator type, and findings were synthesized descriptively by comparing the direction and magnitude of effect across studies for duration of analgesia and time to first rescue analgesic. No pooled effect estimates, heterogeneity statistics, or meta-regression analyses were performed.

Certainty of Evidence Assessment

Certainty of evidence for the primary outcome (duration of postoperative analgesia/time to first rescue analgesic) was evaluated using GRADE principles [11]. Evidence was downgraded for inconsistency due to heterogeneity in buprenorphine dosing regimens and variability in comparator strategies across trials [4-8]. Imprecision was also considered, as several studies had modest sample sizes and were not powered to detect rare adverse events. Risk of bias was judged as low or with some concerns across studies, and no serious indirectness was identified. Publication bias could not be formally assessed due to the limited number of included trials.

Results

Study Selection

**Figure 1:**
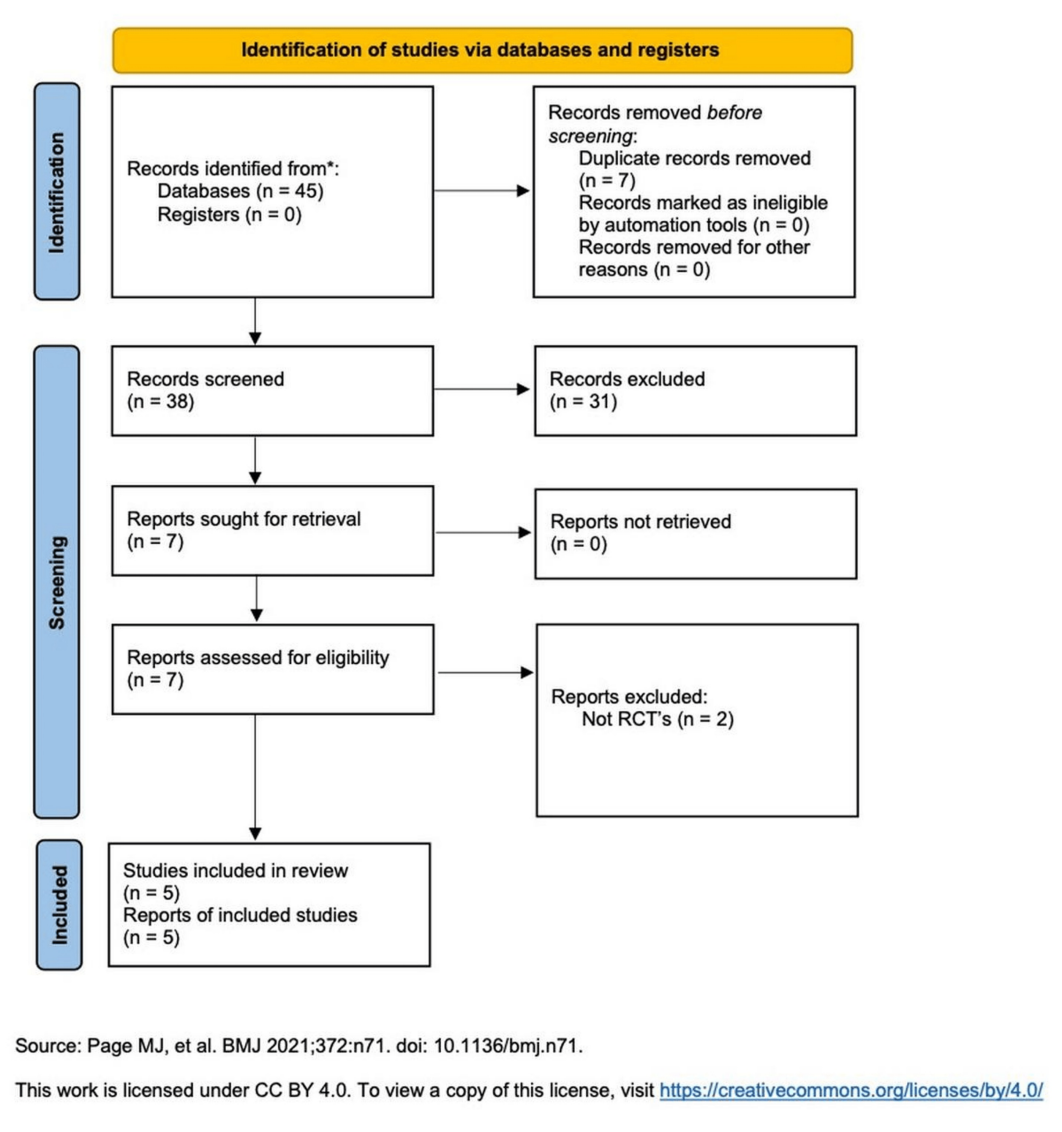
PRISMA flow diagram of study screening and selection PRISMA: Preferred Reporting Items for Systematic Reviews

The database search identified records through PubMed and Cochrane CENTRAL. After removal of duplicates and screening of titles and abstracts, potentially eligible studies underwent full-text review. Five randomized controlled trials met the inclusion criteria [4-8]. Two full-text articles were excluded due to a non-randomized design or failure to meet inclusion criteria. See the PRISMA flow diagram in Figure 1.

Study Characteristics

Five randomized controlled trials involving a total of 727 participants were included (Table 1) [4-8]. Sample sizes ranged from 45 to 442 participants per study. Intrathecal buprenorphine doses ranged from 30 µg to 150 µg. Comparator groups included local anesthetic alone, varying doses of intrathecal buprenorphine, and epidural buprenorphine administration.

**Table 1:** Characteristics of included randomized controlled trials evaluating intrathecal buprenorphine for cesarean delivery

Study	Year	Country	Study Design	Total Sample Size (N)	Buprenorphine Dose (Intrathecal)	Comparator Group	Primary Analgesic Outcome	Duration of Analgesia (Buprenorphine vs Comparator)	Reported Adverse Events	Neonatal Outcomes
Celleno & Capogna (1989) [4]	1989	Italy	Randomized controlled trial (dose-ranging)	45	30–45 µg	Local anesthetic alone	Duration of postoperative analgesia	Dose-dependent prolongation; higher doses achieved significantly longer analgesia (p < 0.01)	Mild nausea; minimal sedation	No adverse neonatal effects reported
Dixit (2007) [5]	2007	India	Prospective randomized controlled trial	60	60 µg	Bupivacaine alone	Duration of analgesia/time to first rescue analgesic	491 ± 153 min vs 145 ± 25 min (p < 0.001)	Vomiting 10%; urinary retention 13.33%; nausea (20%); drowsiness (56%)	Stable Apgar scores
Ravindran et al. (2017) [6]	2017	India	Randomized controlled trial (three-arm)	90	45 µg and 60 µg	Bupivacaine alone	Time to first rescue analgesic	6.1 h (45 µg), 12.3 h (60 µg) vs 2.7 h control (p < 0.05)	Mild sedation; pruritus; nausea and vomiting	No neonatal compromise reported
Ipe et al. (2010) [7]	2010	India	Randomized controlled trial	90	150 µg	Epidural buprenorphine (150 µg / 300 µg)	Duration of analgesia	Intrathecal administration significantly prolonged analgesia compared to the same dose of epidural administration (p < 0.05)	Pruritus; mild nausea	Comparable Apgar scores
Rabiee et al. (2014) [8]	2014	Iran	Double-blind randomized controlled trial	442	100 µg	Control group (local anesthetic)	Duration of postoperative analgesia	18.7 h vs 1.25 h (p < 0.001)	Mild nausea; pruritus	No significant differences in Apgar scores

Primary Outcome: Duration of Analgesia/Time to First Rescue Analgesic

All five randomized controlled trials demonstrated significant prolongation of postoperative analgesia with intrathecal buprenorphine compared with control groups [4-8].

Celleno and Capogna reported dose-dependent prolongation of analgesia across multiple buprenorphine dosing groups [4]. Dixit demonstrated a mean duration of analgesia of 491 ± 153 minutes in the buprenorphine group compared with 145 ± 25 minutes in controls (p < 0.001) [5]. Ravindran et al. observed a graded increase in time to first rescue analgesic with increasing doses of buprenorphine (45 µg versus 60 µg) (p < 0.05) [6]. Ipe et al. found 150 µg intrathecal administration superior compared to 150 µg epidural administration, specifically noting prolonging postoperative analgesia in the intrathecal group (p < 0.05) [7]. Rabiee et al. reported a prolonged analgesic duration of approximately 18.7 hours compared with 1.25 hours in the control group (p < 0.001) [8].

Across trials, analgesic duration ranged from approximately 173 minutes to 18 hours, depending on dose and comparator regimen [4-8].

Secondary Outcomes

Pain scores: Most studies reported significantly lower early postoperative visual analog scale (VAS) pain scores in the intrathecal buprenorphine groups compared with controls [5,6,8].

Rescue analgesic requirements: Reduced supplemental analgesic consumption was consistently reported in buprenorphine groups across trials [5-8].

Adverse events: Adverse events reported included nausea, vomiting, pruritus, and mild sedation [4-8]. While lower doses (≤60 µg) were associated with fewer adverse effects, higher doses (≥100 µg) were more frequently associated with increased sedation or pruritus [6,7], although no clinically significant respiratory depression was reported in any included study [4-8].

Neonatal outcomes: Among studies reporting neonatal outcomes, Apgar scores were comparable between buprenorphine and control groups [5,7,8].

Risk of Bias Assessment

Overall, the included studies were judged to have a low risk of bias or some concerns according to the Cochrane Risk of Bias 2 tool. See Table 2 for the risk of bias assessment. Older trials demonstrated incomplete reporting of allocation concealment procedures [4], whereas more recent studies provided clearer descriptions of randomization and outcome assessment methods [6-8]. No study was judged to be at high risk of bias across multiple domains for the primary analgesic outcome.

**Table 2:** Risk of bias assessment of included randomized controlled trials using the Cochrane RoB 2 tool (primary analgesic outcome)

Study	Randomization Process	Deviations from Intended Interventions	Missing Outcome Data	Measurement of Outcome	Selection of Reported Results	Overall Risk of Bias
Celleno & Capogna (1989) [4]	Some concerns	Low	Low	Low	Some concerns	Some concerns
Dixit (2007) [5]	Some concerns	Low	Low	Low	Some concerns	Some concerns
Ravindran et al. (2017) [6]	Low	Low	Low	Low	Some concerns	Some concerns
Ipe et al. (2010) [7]	Low	Low	Low	Low	Some concerns	Some concerns
Rabiee et al. (2014) [8]	Low	Low	Low	Low	Some concerns	Some concerns

Certainty of Evidence

Certainty of evidence for the primary outcome of prolonged postoperative analgesia was rated as low to moderate according to GRADE principles [11]. Evidence was downgraded for inconsistency due to heterogeneity in buprenorphine dosing regimens and variability in comparator strategies across trials [4-8]. Imprecision was also considered, as several studies had modest sample sizes and were not powered to detect rare adverse events. Risk of bias was judged as low or with some concerns across studies, and no serious indirectness was identified. Publication bias could not be formally assessed due to the limited number of included trials.

Discussion

This systematic review synthesizes randomized evidence evaluating the efficacy and safety of intrathecal buprenorphine for postoperative analgesia following cesarean delivery. Across five randomized controlled trials involving 727 participants, intrathecal buprenorphine consistently prolonged postoperative analgesia and delayed time to first rescue analgesic compared with control regimens [4-8]. The direction of effect was uniform across all included studies, supporting a consistent analgesic benefit.

A key finding across trials was a dose-dependent prolongation of analgesia [4-6]. From a clinical perspective, lower doses may provide a favorable balance between analgesic efficacy and reduced incidence of adverse effects. Lower doses (45-60 µg) provided clinically meaningful extension of postoperative analgesia in the range of approximately 6 to 12 hours [5,6], whereas higher doses (100-150 µg) achieved durations approaching 18 hours in some studies [7,8]. Celleno and Capogna demonstrated a clear dose-response relationship in their multi-arm trial design [4], and Ravindran et al. similarly reported graded prolongation of analgesia with increasing doses [6]. Despite differences in comparator groups, including local anesthetic alone and epidural buprenorphine, the magnitude and direction of benefit remained consistent [4-8].

The observed dose-response relationship is biologically plausible given buprenorphine’s pharmacologic properties. As a partial μ-opioid receptor agonist with high receptor affinity and slow dissociation kinetics, buprenorphine exhibits prolonged receptor occupancy within the spinal cord [3]. Its lipid solubility may facilitate effective spinal receptor binding while potentially limiting extensive cephalad cerebrospinal fluid migration. This is in contrast to hydrophilic opioids, such as morphine, which exhibit greater cephalad cerebrospinal fluid spread and are associated with delayed respiratory depression; lipophilic agents, such as buprenorphine, demonstrate more limited rostral migration, although this effect is not absolute and may vary with dose. These pharmacodynamic characteristics likely contribute to the sustained analgesic effects observed across trials.

In addition to prolonging time to first analgesic request, intrathecal buprenorphine was associated with reduced supplemental analgesic consumption in studies reporting this outcome [5-8]. Reduced need for additional opioids or nonsteroidal anti-inflammatory drugs was consistently reported across studies; however, the magnitude of reduction could not be quantitatively pooled due to heterogeneity in reporting. These findings nonetheless suggest that the prolongation of analgesia translated into clinically meaningful reductions in breakthrough pain rather than simply a statistical delay in rescue medication timing.

The safety profile observed across included trials was generally favorable. Reported adverse events were mild and consistent with known opioid effects, including nausea, vomiting, pruritus, and sedation [4-8]. Importantly, no clinically significant respiratory depression was reported in any included study [4-8]. Delayed respiratory depression is a recognized concern with intrathecal morphine due to cephalad cerebrospinal fluid migration and medullary opioid receptor interaction [2]. Although the included buprenorphine trials were not powered to detect rare respiratory events, the absence of reported clinically significant ventilatory compromise across studies is reassuring. Nevertheless, given modest sample sizes, rare adverse events cannot be definitively excluded.

Higher buprenorphine doses were occasionally associated with increased sedation or pruritus [6,7], indicating that while analgesic duration may increase with dose escalation, adverse effects may also rise proportionally. Determination of optimal dosing, therefore, requires balancing analgesic efficacy with tolerability.

Intrathecal morphine remains widely regarded as the reference neuraxial opioid for cesarean delivery due to its prolonged analgesic duration and established use in clinical practice [2]. While direct head-to-head randomized comparisons between intrathecal buprenorphine and intrathecal morphine were not included among eligible trials, higher-dose buprenorphine regimens demonstrated analgesic durations approaching those typically associated with intrathecal morphine [7,8]. However, the absence of standardized comparative trials limits definitive conclusions regarding equivalence or superiority. Direct randomized comparisons using uniform dosing and outcome definitions are needed to clarify relative efficacy and safety.

Several methodological considerations warrant discussion. Heterogeneity across included trials was present in buprenorphine dosing (30-150 µg), comparator regimens, and timing of outcome assessment [4-8]. Older trials demonstrated less detailed reporting of allocation concealment procedures [4], whereas more recent studies provided clearer methodological descriptions [6-8]. Despite these variations, overall risk of bias was assessed as low or with some concerns, and no study was judged to be at high risk across multiple domains for the primary analgesic outcome.

The small number of eligible trials and variability in outcome reporting precluded quantitative meta-analysis. A narrative synthesis was therefore undertaken. While consistency in the direction of effect strengthens confidence in analgesic benefit, standardized dosing regimens and outcome definitions would enhance future quantitative synthesis.

Limitations and future directions

Despite the overall consistency of findings, several limitations warrant consideration. Only five randomized controlled trials met strict inclusion criteria, limiting statistical power and generalizability. Variability in dosing strategies and comparator regimens restricted the ability to perform meta-analysis. Although a protocol was developed prior to study selection, the review was not prospectively registered in a public registry. Additionally, the included studies were not powered to detect rare adverse events such as clinically significant respiratory depression.

Future research should prioritize direct head-to-head randomized trials comparing standardized doses of intrathecal buprenorphine and intrathecal morphine. Larger sample sizes, uniform outcome definitions, and standardized reporting of adverse events would enhance the strength of evidence. Evaluation within enhanced recovery pathways may further clarify the role of intrathecal buprenorphine in contemporary obstetric anesthesia practice.

## Conclusions

Intrathecal buprenorphine appears to provide clinically meaningful prolongation of postoperative analgesia following cesarean delivery, with consistent delay in time to first rescue analgesic across randomized controlled trials. A dose-dependent relationship was observed, with higher doses associated with longer analgesic duration but potential increases in mild opioid-related adverse effects. Across included studies, adverse events were generally limited to nausea, vomiting, pruritus, and sedation, and no clinically significant respiratory depression was reported. However, modest sample sizes and heterogeneity in dosing regimens limit the certainty of conclusions.

Although intrathecal morphine remains the most commonly used neuraxial opioid for cesarean delivery, intrathecal buprenorphine may represent a viable alternative in selected clinical settings. Larger, well-designed randomized trials directly comparing standardized doses of intrathecal buprenorphine and intrathecal morphine are warranted to clarify relative efficacy and safety.
